# Redução na Hospitalização e Aumento na Mortalidade por Doenças Cardiovasculares durante a Pandemia da COVID-19 no Brasil

**DOI:** 10.36660/abc.20200821

**Published:** 2021-02-04

**Authors:** Paulo Garcia Normando, José de Arimatéia Araujo-Filho, Gabriela de Alcântara Fonseca, Rodrigo Elton Ferreira Rodrigues, Victor Agripino Oliveira, Ludhmila Abrahão Hajjar, André Luiz Cerqueira Almeida, Edimar Alcides Bocchi, Vera Maria Cury Salemi, Marcelo Melo

**Affiliations:** 1 Universidade Federal da Paraíba João PessoaPB Brasil Universidade Federal da Paraíba , João Pessoa , PB - Brasil; 2 Universidade de São Paulo Instituto do Coração - Centro de Diagnóstico por Imagem São PauloSP Brasil Universidade de São Paulo Instituto do Coração - Centro de Diagnóstico por Imagem , São Paulo , SP - Brasil; 3 Hospital Sírio Libanês São PauloSP Brasil Hospital Sírio Libanês , São Paulo , SP - Brasil; 4 Instituto de Coração – Cardiopneumologia São PauloSP Brasil Instituto de Coração – Cardiopneumologia , São Paulo , SP - Brasil; 5 Santa Casa de Misericórdia de Feira de Santana Feira de SantanaBA Brasil Santa Casa de Misericórdia de Feira de Santana – Cardiologia, Feira de Santana , BA - Brasil

**Keywords:** COVID-19, Betacoronavírus, Pandemia, Doenças Cardiovasculares/complicações, Epidemiologia, Hospitalização, Mortalidade, Comorbidades, Sistema Único de Saúde (SUS)

## Abstract

**Fundamento:**

Na pandemia pela COVID-19, o aumento da ocorrência e da mortalidade por doenças cardiovasculares (DCV) vem sendo reconhecido no mundo. No Brasil, é essencial que o impacto da COVID-19 na DCV seja analisado.

**Objetivos:**

Avaliar o impacto desta pandemia nos números de internações hospitalares (IH), óbitos hospitalares (OH) e letalidade intra-hospitalar (LH) por DCV a partir de dados epidemiológicos do Sistema Único de Saúde (SUS).

**Métodos:**

Estudo observacional de séries temporais por meio da análise comparativa das taxas de IH, OH e LH por DCV registrados entre janeiro e maio de 2020, usando como referência os valores obtidos no mesmo período entre 2016 e 2019 e os valores projetados por métodos de regressão linear para o ano de 2020. O nível significância estatística utilizado foi de 0,05.

**Resultados:**

Em comparação com o mesmo período de 2019, houve um decréscimo de 15% na taxa de IH e de 9% no total de OH por DCV entre março e maio de 2020, acompanhado de um aumento de 9% na taxa de LH por esse grupo de doenças, sobretudo entre pacientes com idade de 20-59 anos. As taxas de IH e LH registradas em 2020 diferiram significativamente da tendência projetada para o corrente ano (p=0,0005 e 0,0318, respectivamente).

**Conclusões:**

Durante os primeiros meses da pandemia, observou-se um declínio na IH associado a um aumento da LH por DCV no Brasil. Esses dados possivelmente são consequência do planejamento inadequado no manejo das DCV durante a pandemia, sendo necessária a implementação de ações imediatas para modificar esse cenário. (Arq Bras Cardiol. 2021; [online].ahead print, PP.0-0)

## Introdução

A COVID-19 (sigla do inglês *Coronavirus Disease* - 2019) foi declarada uma pandemia pela Organização Mundial da Saúde em 11 de março de 2020. Em julho de 2020, o Brasil era o segundo país do mundo em número de casos e mortes por COVID-19. Dados do dia 24 de julho de 2020 registraram 2.276.860 casos confirmados da doença no país, responsável por 84.551 mortes. ^1^

Considerando que a transmissão se dá principalmente por gotículas expelidas durante a fala, tosse e espirro ou pela contaminação de superfícies, ^4^ restrições à circulação e contato entre pessoas foram propostas pelas autoridades governamentais e de saúde pública na maioria dos países ocidentais. No Brasil, os desafios logísticos para atender à demanda de pacientes incluíram a ampliação de leitos em unidades de terapia intensiva e enfermarias, a suspensão de atendimentos e da realização de exames complementares e de procedimentos eletivos, bem como o direcionamento dos recursos públicos para o atendimento dessa doença. ^5^

Estudos populacionais em outros países registraram uma redução relativa nas admissões hospitalares por doenças cardiovasculares (DCV) durante a corrente pandemia, ^8 , 9^ associada a um aumento nas taxas de letalidade previamente relacionadas a esse grupo de doenças, ^10 , 11^ motivo de grande preocupação entre a comunidade médica e científica internacional.

Diante do exposto, testamos a hipótese de que, durante a pandemia da COVID-19, um menor número de atendimentos e de intervenções cardiovasculares tenha ocorrido e tal fato poderia resultar em uma maior letalidade intra-hospitalar por DCV na população geral. Utilizando bases de dados de domínio público do Sistema Único de Saúde (SUS), o presente estudo teve o objetivo de avaliar o impacto da pandemia nos números de internações hospitalares e na letalidade intra-hospitalar por DCV no território nacional entre janeiro e maio de 2020, em comparação com o mesmo período dos quatro anos anteriores, englobando a busca de elementos clínicos pré-hospitalares, incluindo procedimentos eletivos, que corroboram essas evidências.

## Métodos

Este é um estudo observacional de séries temporais, que compreende as internações, óbitos hospitalares, taxa de letalidade intra-hospitalar (percentual de óbitos dentre as internações) e procedimentos hospitalares e ambulatoriais realizados – sendo os dois primeiros divididos nos grupamentos infanto-juvenil (faixa etária de 0 até 19 anos), adulto (20 a 59 anos) e idosos (60 anos ou mais) – em razão de condições relacionadas ao sistema cardiovascular nos serviços próprios e conveniados ao SUS entre janeiro e maio dos anos de 2016 a 2020. Os dados foram coletados em 9 de julho de 2020 mediante busca nos Sistemas de Informações Hospitalares e Ambulatoriais do SUS (SIH e SIA/SUS, respectivamente), disponíveis na plataforma DATASUS e, portanto, públicos e anônimos em conformidade com o artigo I da resolução 510/2016 da Comissão Nacional de Ética em Pesquisa. Vale ressaltar que atualizações de internações passadas podem ocorrer na plataforma e, portanto, não há como se certificar que todos os dados estão consolidados, independentemente do ano.

Para a análise dos procedimentos, foram utilizados os códigos do Sistema de Gerenciamento da Tabela de Procedimentos, Medicamentos e Órteses, Próteses e Materiais do SUS (SIGTAP). Foram somadas as produções hospitalar e ambulatorial de cada procedimento selecionado, considerando todos os códigos de procedimento correlacionados, sendo eles:

Procedimentos diagnósticos: cateterismo cardíaco, ecocardiografia (de estresse, transesofágica e transtorácica), eletrocardiograma, implante de marca-passo cardíaco, monitoramento pelo sistema Holter 24 horas, monitorização ambulatorial de pressão arterial ( MAPA ), teste de esforço/ergométrico.

Procedimentos cirúrgicos: cirurgias cardiovascular, endovascular e vascular.

Para o número de internações, óbitos hospitalares e letalidade intra-hospitalar, foram selecionados, para cada uma das três faixas etárias, os registros dos diagnósticos secundários relacionados a patologias cardiovasculares correspondentes à Lista de Morbidade da Décima Revisão da Classificação Estatística Internacional de Doenças e Problemas Relacionados à Saúde (CID-10). As seguintes categorias de doenças foram consideradas: acidente vascular cerebral (AVC), doenças hipertensivas (hipertensão arterial essencial e outras doenças hipertensivas), doenças reumáticas (febre reumática aguda e doença reumática crônica do coração), infarto agudo do miocárdio (IAM), insuficiência cardíaca, malformações congênitas do aparelho circulatório e transtornos de condução e arritmias.

### Análise Estatística

Uma vez que os dados de procedimentos e cirurgias estão discriminados no sistema apenas quanto à sua natureza (ambulatorial ou hospitalar), uma análise descritiva de como tais procedimentos se dividiam nessas categorias foi realizada. Com relação aos dados de internação e óbitos hospitalares, variáveis como gênero, faixa etária, cor/raça e caráter de atendimento (urgência ou emergência) foram consideradas.

Tendo como objetivo entender o possível impacto da pandemia na dinâmica dos procedimentos, internações e óbitos hospitalares relacionados às DCV, foram comparados os montantes relativos aos meses de março, abril e maio – meses mais afetados no presente ano pela pandemia no Brasil – para os anos de 2016 a 2020. Foram confrontados os valores registrados entre 2019 e 2020 e a variação percentual correspondente entre eles. Vale ressaltar que uma variação na quantidade de internações e óbitos hospitalares entre um ano e outro ou uma mudança na média dos anos anteriores com relação ao ano atual não é causada necessariamente pela pandemia. Consideramos essa variação hipotética uma possível consequência de uma tendência já estabelecida nos anos anteriores. Finalmente, o valor esperado para o ano de 2020 foi estimado, por meio de regressão linear. Tal análise permite presumir se a quantidade de procedimentos, de cirurgias, de internações e de óbitos hospitalares ou a taxa de letalidade intra-hospitalar que vinha sendo observada nos anos anteriores a 2020 apresentava uma tendência de crescimento ou redução. Portanto, a análise realizada capturou tanto a tendência dos anos quanto as variações estatísticas que ocorreram nos anos anteriores.

Embora testes de normalidade não tenham sido realizados, assumiu-se a normalidade dos dados ao longo do tempo considerando-se o teorema central do limite, visto que o dado de cada ano é uma totalização de diversas variáveis aleatórias. A homocedasticidade não pode ser completamente verificada, uma vez que o DATASUS não fornece os dados inteiramente individualizados.

Tais análises estatísticas foram realizadas tanto com o montante de cirurgias e procedimentos, como individualmente para cada procedimento/cirurgia. Da mesma maneira, essas análises foram feitas para internações, óbitos hospitalares e letalidade intra-hospitalar, tanto considerando cada morbidade estudada individualmente como considerando a soma de todas elas, ou seja, uma análise das causas cardiovasculares de uma forma geral.

Como a regressão linear apresenta um erro gaussiano, foi realizado o teste *t* de Student para média de uma amostra de modo a comparar os valores projetados com os registrados em 2020, sendo rejeitada a hipótese nula com um p<0,05 (intervalo de confiança de 95%). Os programas Microsoft® Excel® e Scilab® 6.1.0 foram utilizados para realizar as análises estatísticas descritas e confecção das tabulações e gráficos.

## Resultados

### Análise Descritiva

A partir dos dados coletados referentes aos meses de janeiro a maio dos anos de 2016 a 2020, foram levantados 35.744.058 procedimentos, 1.336.472 internações e 142.157 óbitos hospitalares, sendo os dois últimos divididos por região, gênero, faixa etária, raça/cor e caráter de atendimento conforme dados demonstrados na Tabela 1 . Os dados completos que ilustram a variação dos números iniciando em março de 2020 são apresentados nas figuras do material suplementar.

**Tabela 1:** – Análise descritiva do número de procedimentos, cirurgias, internações e óbitos hospitalares nos meses de janeiro a maio dos anos de 2016 a 2020

	HOSPITALARES		AMBULATORIAIS	
	Quantidade	Percentual	Quantidade	Percentual
**Procedimentos**				
Cateterismo adulto	209.926	3,66%	252.162	0,87%
Ecocardiografia	989.776	17,24%	2.751.796	9,53%
Eletrocardiograma	4.461.799	77,71%	21.587.674	74,75%
Implante de marca-passo	48.666	0,85%	-	-
MAPA	-	-	2.763.748	9,57%
Monitoramento Holter	26.119	0,45%	513.471	1,78%
Teste ergométrico	5.630	0,10%	1.010.144	3,50%
**Total**	**5.741.916**	**100,00%**	**28.878.995**	**100,00%**
**Cirurgias**				
Cardiovascular	193.787	36,45%	-	-
Endovascular	48.024	9,03%	-	-
Vascular	289.815	54,51%	591.521	100,00%
**Total**	**531.626**	**100,00%**	**591.521**	**100,00%**
**Procedimentos**				
Região				
Norte	179.761	3,13%	1.442.382	4,99%
Nordeste	896.916	15,62%	5.551.764	19,22%
Sudeste	3.071.586	53,49%	15.271.343	52,88%
Sul	1.236.067	21,53%	4.285.803	14,84%
Centro-Oeste	357.586	6,23%	2.327.703	8,06%
**Brasil**	**5.741.916**	**100,00%**	**28.878.995**	**100,00%**
**Cirurgias**				
Região				
Norte	13.676	2,57%	13.774	2,33%
Nordeste	98.475	18,52%	442.540	74,81%
Sudeste	239.747	45,10%	81.777	13,82%
Sul	140.536	26,44%	43.469	7,35%
Centro-Oeste	39.192	7,37%	9.961	1,68%
Brasil	531.626	100,00%	591.521	100,00%
	**INTERNAÇÕES**		**ÓBITOS**	
	**Quantidade**	**Percentual**	**Quantidade**	**Percentual**
**Morbidade**				
AVC	357.040	26,72%	52.239	36,75%
Doenças Hipertensivas	153.048	11,45%	2.920	2,05%
Doenças Reumáticas	19.125	1,43%	1.329	0,93%
IAM	242.143	18,12%	24.753	17,41%
Insuficiência cardíaca	399.416	29,89%	43.906	30,89%
Malformações congênitas	33.939	2,54%	2.370	1,67%
Transtornos de condução e arritmias	131.761	9,86%	14.640	10,30%
**Total**	**1.336.472**	**100,00%**	**142.157**	**100,00%**
**Região**				
Norte	77.577	5,80%	8.578	6,03%
Nordeste	327.515	24,51%	35.749	25,15%
Sudeste	563.293	42,15%	63.609	44,75%
Sul	270.995	20,28%	23.624	16,62%
Centro-Oeste	97.092	7,26%	10.597	7,45%
**Total**	**1.336.472**	**100,00%**	**142.157**	**100,00%**
**Sexo**				
Masculino	704.163	52,69%	73.227	51,51%
Feminino	632.309	47,31%	68.930	48,49%
**Total**	**1.336.472**	**100,00%**	**142.157**	**100,00%**
**Raça/Cor**				
Branca	491.422	36,77%	48.962	34,44%
Preta	58.968	4,41%	6.301	4,43%
Parda	435.848	32,61%	46.276	32,55%
Amarela	28.768	2,15%	2.764	1,94%
Indígena	1.025	0,08%	107	0,08%
Sem informação	320.441	23,98%	37.747	26,55%
**Total**	**1.336.472**	**100,00%**	**142.157**	**100,00%**
**Faixa etária (anos)**				
0-19	44.658	3,34%	3.241	2,28%
20-59	415.122	31,06%	29.245	20,57%
60+	876.692	65,60%	109.671	77,15%
**Total**	**1.336.472**	**100,00%**	**142.157**	**100,00%**
**Caráter de atendimento**				
Eletivo	104.229	7,80%	5.454	3,84%
Urgência	1.232.243	92,20%	136.703	96,16%
**Total**	**1.336.472**	**100,00%**	**142.157**	**100,00%**

*AVC: acidente vascular cerebral; IAM: infarto agudo do miocárdio; MAPA: medição ambulatorial da pressão arterial.*

### Análise Gráfica

**Figura 1:**
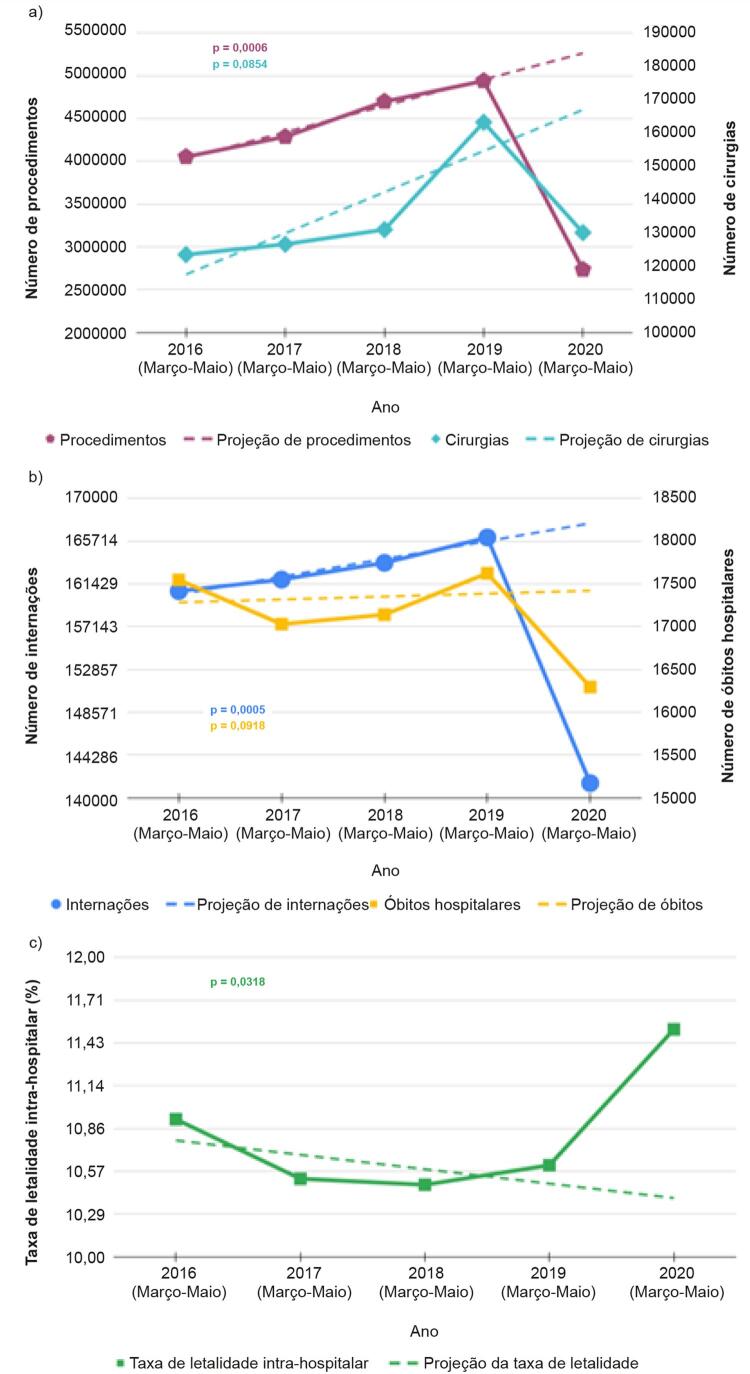
– Análise da tendência do número de (a) procedimentos, cirurgias; (b) internações, óbitos; e (c) taxa de letalidade intra-hospitalar nos meses de março a maio dos anos de 2016 a 2020. p-valor calculado a partir da diferença entre o valor projetado e o registrado em 2020 utilizando a distribuição t de Student.

A Figura 1A apresenta os dados relativos aos procedimentos diagnósticos e cirúrgicos pesquisados entre os anos 2016 e 2020, para os meses de março a maio. Também são expostas as projeções para 2020 que foram calculadas utilizando-se os dados de 2016 a 2019. Nota-se uma tendência de crescimento do número de procedimentos diagnósticos e cirúrgicos (linhas pontilhadas) para o ano de 2020. No entanto, os dados reais mostram uma queda expressiva quando comparados aos realizados no ano anterior.

A Figura 1B apresenta a quantidade de internações e óbitos hospitalares registrados, considerando todas as morbidades pesquisadas. Também são expostas as projeções para 2020, que foram calculadas utilizando os dados de 2016 a 2019. O comportamento observado é semelhante ao do gráfico anterior, onde existe uma tendência de crescimento não confirmada no ano de 2020, quando se observa uma queda acentuada. Com relação ao número de óbitos, a tendência projetada seria de manutenção, o que contrasta com a intensa redução no número de óbitos hospitalares registrados nos meses de março a maio de 2020.

A Figura 1C destaca a taxa de letalidade intra-hospitalar geral por DCV registrada. Pode ser percebido um drástico aumento na taxa de letalidade intra-hospitalar em 2020 se comparado aos números registrados nos anos anteriores. Nesse caso, a direção da variação é oposta às observadas para internações e óbitos hospitalares, que apresentaram decréscimo em 2020.

Tal análise foi replicada para cada tipo de procedimento e cirurgia, assim como para internações, óbitos hospitalares e taxa de letalidade intra-hospitalar em cada patologia estudada. Esses resultados são sumarizados nas Tabelas 2 e 3 , apresentando o registro de 2019 e 2020 para a diferença percentual entre esses anos, o valor projetado para 2020 (que indica a tendência de 2016 a 2019), o intervalo de confiança e o valor de p dessa projeção. Os resultados detalhados por faixa etária são apresentados no material suplementar do artigo.

**Tabela 2:** – Análise estatística da redução do número de procedimentos e cirurgias nos meses de março a maio comparando-se os anos de 2019 e 2020

	Quantidade de procedimentos		Diferença percentual 2020 - 2019	Quantidade de procedimentos estimados em 2020 (março-maio)	Intervalo de confiança (IC 95%)	p-valor
	2019 março-maio	2020 março-maio				
**Procedimentos**						
Cateterismo adulto	61.502	44.652	-27%	63.823	68.921 – 58.724	0,0041
Ecocardiografia	530.448	307.221	-42%	568.014	599.663 – 536.364	0,0009
Eletrocardiograma	3.624.680	2.153.969	-41%	3.795.712	3.952.567 – 3.638.856	0,0005
Implante de marca-passo	5.993	5.315	-11%	6.129	6.711 – 5.546	0,0276
MAPA	498.923	127.730	-74%	609.445	748.875 – 470.014	0,0048
Monitoramento Holter	79.792	38.815	-51%	84.592	93.971 – 75.212	0,0024
Teste ergométrico	137.678	56.501	-59%	134.673	149.970 – 119.375	0,0022
**Todos**	**4.939.016**	**2.734.203**	**-45%**	**5.262.388**	**5.516.598 – 5.008.177**	**0,0006**
**Cirurgias**						
Cardiovascular	23.907	20.744	-13%	24.045	26.897 – 21.191	0,0388
Vascular	101.694	83.786	-18%	104.654	159.586 – 49.720	0,1914
Endovascular	37.547	25.461	-32%	38.208	45.145 – 31.269	0,0165
**Todas **	**163.148**	**129.991**	**-20%**	**166.906**	**218.270 – 115.540**	**0,0854**
**TOTAL**	**5.102.164**	**2.864.194**	**-44%**	**5.429.294**	**5.670.710 – 5.187.876**	**0,0005**

*MAPA: medição ambulatorial da pressão arterial. Intervalos de confiança e p-valor calculados utilizando a distribuição t de Student, considerando as diferenças entre o valor projetado e o registrado em 2020.*

**Tabela 3:** – Análise estatística do número de internações, óbitos hospitalares e taxa de letalidade intra-hospitalar nos meses de março a maio comparando-se os anos de 2019 e 2020

	Dados registrados em		Diferença percentual 2020 - 2019	Estimativas para 2020 março-maio	Intervalo de confiança (IC 95%)	p-valor
	2019 março - maio	2020 março - maio				
**Internações**						
Acidente vascular cerebral	45.214	39.900	-12%	46.199	48.586 – 43.811	0,0082
Doenças hipertensivas	18.278	12.229	-33%	18.053	20.331 – 15.773	0,0088
Doenças reumáticas	2.403	1.701	-29%	2.266	2.683 – 1.847	0,0294
Infarto agudo do miocárdio	31.566	30.298	-4%	33.084	35.550 – 30.616	0,0405
Insuficiência cardíaca	47.250	39.667	-16%	46.077	49.847 – 42.305	0,0191
Malformações cardiovasculares	4.489	3.692	-18%	4.602	4.883 – 4.320	0,0055
Transtornos de condução e arritmias cardíacas	16.875	13.977	-17%	17.212	18.316 – 16.107	0,0067
** Comorbidades cardiovasculares**	**166.075**	**141.464**	**-15%**	**167.491**	**169.772 – 165.209**	**0,0005**
**Óbitos hospitalares**						
Acidente vascular cerebral	6411	5871	-8%	6363	7364 - 5361	0,1439
Doenças hipertensivas	336	289	-14%	309	361 - 256	0,1926
Doenças reumáticas	180	144	-20%	161	262 - 58	0,3414
Infarto agudo do miocárdio	3081	2805	-9%	3094	3500 - 2686	0,0872
Insuficiência cardíaca	5356	4845	-10%	5212	5612 - 4811	0,0579
Malformações cardiovasculares	307	288	-6%	297	382 - 210	0,3995
Transtornos de condução e arritmias cardíacas	1952	2053	5%	1986	2582 - 1388	0,3863
** Comorbidades cardiovasculares**	**17623**	**16295**	**-8%**	**17420**	**19062 - 15777**	**0,0918**
**Taxa de letalidade intra-hospitalar**						
Acidente vascular cerebral	14,18	14,71	4%	13,72%	15,33 - 12,10	0,1067
Doenças hipertensivas	1,84	2,36	29%	1,72%	2,11 - 1,34	0,0198
Doenças reumáticas	7,49	8,47	13%	7,08%	10,19 - 3,97	0,1616
Infarto agudo do miocárdio	9,76	9,26	-5%	9,20%	10,21 - 8,18	0,4363
Insuficiência cardíaca	11,34	12,21	8%	11,31%	12,01 - 10,60	0,0322
Malformações cardiovasculares	6,84	7,80	14%	6,40%	8,26 - 4,54	0,0798
Transtornos de condução e arritmias cardíacas	11,57	14,69	27%	11,57%	14,90 - 8,24	0,0559
** Comorbidades cardiovasculares**	**10,61**	**11,52**	**9%**	**10,39%**	**11,26 - 9,52**	**0,0318**

*AVC: acidente vascular cerebral; IAM: infarto agudo do miocárdio; Intervalos de confiança e p-valor calculados utilizando a distribuição t de Student, considerando as diferenças entre o valor projetado e o registrado em 2020.*

#### Procedimentos Diagnósticos e Cirúrgicos

A Tabela 2 apresenta a comparação do número de procedimentos diagnósticos e cirúrgicos realizados nos meses de março, abril e maio dos anos de 2019 e 2020, percebendo-se uma queda total de 45% em todos os procedimentos pesquisados no ano de 2020. Os procedimentos que tiveram diminuição mais expressiva foram: MAPA (redução de 74%), teste ergométrico (59%) e Holter 24hs (51%). Eletrocardiograma e ecocardiograma apresentaram queda de 41% e 42%, respectivamente. Os que apresentaram menor declínio foram o cateterismo e o implante de marca-passo, com redução de 27% e 11%, respectivamente.

O número total de cirurgias apresentou uma queda de 20% com relação ao ano anterior, o que não foi estatisticamente significante (p=0,0854). No entanto, quando se consideram apenas as cirurgias cardio- e endovasculares, quedas de 13% e 32% foram registradas, ambas com significância estatística (p<0,05).

## Internações

Quanto ao número de internações hospitalares por razões cardiovasculares, foi registrada, nos meses de março, abril e maio de 2020, uma redução de 15% em relação ao mesmo período do ano anterior ( Tabela 3 ). Os dados de todas as doenças analisadas individualmente também apresentaram redução estatisticamente significante. As maiores diferenças foram internações por doenças hipertensivas, seguidas pelas reumáticas, com queda de 33% e 29%, respectivamente. Destaca-se que o IAM apresentou a menor queda (4%).

De modo geral, todas as doenças apresentaram decréscimo entre os anos 2019 e 2020 para todas as faixas etárias. Destacam-se o IAM, cuja diferença nas internações apresentou significância estatística apenas para o grupo dos idosos, a insuficiência cardíaca, na qual os grupos extremos (infantojuvenil e idosos) foram os mais impactados, e os transtornos de condução e outras arritmias que apresentaram redução estatisticamente significante apenas para os idosos. Ao considerar internações por AVC, os adultos e os idosos apresentaram uma redução significativa (11% e 12%, respectivamente). Para as internações por doenças hipertensivas, todas as faixas etárias apresentaram diminuição estatisticamente significante.

## Óbitos Hospitalares e Taxa de Letalidade Intra-hospitalar

Ao se analisar o número absoluto de mortes por DCV, houve um decréscimo de 8% no total entre março e maio de 2020 em comparação ao mesmo período de 2019 ( Tabela 3 ). Os óbitos ocorridos associados a doenças hipertensivas na faixa etária de 20-59 anos apresentaram um acréscimo de 21% no ano de 2020 (p<0,05; arquivo suplementar).

Já com relação às taxas de letalidade geral, pôde-se observar um aumento global de 9%, comparando-se os mesmos meses de 2019 e 2020. Com exceção do IAM, que apresentou queda de 5%, todas as demais patologias apresentaram crescimento. Com relação às patologias individuais, destacam-se doenças hipertensivas e insuficiência cardíaca, com 29% e 8% de aumento na taxa de letalidade intra-hospitalar, respectivamente, entre 2019 e 2020 (p<0,05).

Na divisão por faixas etárias, o aumento da letalidade intra-hospitalar nas internações por DCV foi estatisticamente significativo apenas entre os adultos. Considerando as doenças individualmente, ressalta-se que esse aumento observado na letalidade intra-hospitalar das doenças hipertensivas foi associado a significância estatística apenas para os adultos; quanto aos dados para insuficiência cardíaca, os adultos e idosos apresentaram diferença estatística (p<0,05; Tabelas 2 e 3 do suplemento).

## Discussão

Nosso estudo demonstra uma redução da assistência à saúde cardiovascular da população brasileira atendida pelo SUS durante o período da pandemia da COVID-19, que teve como consequências a redução do número de internações por DCV e o aumento da taxa de letalidade intra-hospitalar decorrente dessas.

Nossos resultados têm semelhanças com os achados de um estudo realizado na Itália ao longo de sete dias do mês de março de 2020, que demonstrou que a proporção de pacientes com IAM foi reduzida em 13,3%, comparada à mesma semana de 2019. Ainda, foi constatado um aumento de 39,2% nas síndromes coronarianas agudas, enquanto o tempo entre o contato médico e a revascularização coronariana aumentou em 31,5%. ^12^ Com relação ao número de internações do nosso estudo, houve redução em todas as morbidades e faixas etárias analisadas, principalmente nos meses de abril e maio, possivelmente um reflexo da pandemia da COVID-19. Outros estudos realizados em diferentes países registraram achados semelhantes. ^13^ O receio da população em contrair o vírus e a sistematização da assistência, priorizando a pandemia, justificam esse impacto inicial. ^16 , 17^ Essa redução também ocorreu para outras doenças, como mostram os estudos com AVC na Itália ^18^ e na China. ^19^

A realocação dos recursos humanos no enfrentamento à COVID-19 foi similar em diversos países, apesar de possuírem uma heterogeneidade em seus sistemas de saúde. ^12^ Países como Austrália e Nova Zelândia, no intuito de preparar o hospital para prestar atendimento a um grande volume de pacientes com COVID-19, mantiveram um fornecimento de atendimentos cirúrgicos limitado a casos de emergência e eletivos de alta prioridade. ^20^ Em hospital da região norte da Itália, um dos epicentros da pandemia no país, as atividades cirúrgicas planejadas foram interrompidas para aumentar o número de intensivistas disponíveis para pacientes com COVID-19, além de as ambulatoriais terem sido reduzidas pela metade. ^21^ Era de se esperar que tal rearranjo no modelo assistencial apresentasse um impacto na mortalidade por outras enfermidades, uma vez que tais medidas reduziram o fluxo habitual de seu atendimento, favorecendo descompensações clínicas, atraso diagnóstico e progressão de doença.

Apesar da redução em números absolutos de óbitos hospitalares, houve aumento da taxa de letalidade intra-hospitalar nas internações por DCV. A redução do número de óbitos pode ser reflexo da falta de notificação adequada, da falta de estrutura do sistema de saúde em atribuir a causa do óbito relacionado à COVID-19 como cardiovascular. A interação COVID-19 e sistema cardiovascular atualmente é bastante reconhecida, após 7 meses da doença no mundo. A COVID-19 está relacionada a alta prevalência de lesão cardíaca, arritmias, miocardite, síndrome coronária aguda, insuficiência cardíaca, choque cardiogênico e eventos tromboembólicos. ^22^ O aumento da letalidade nas internações por DCV reflete o potencial de gravidade da COVID-19 nas DCV e possivelmente o retardo do paciente em procurar assistência médica, sendo admitido no hospital em condição mais grave. O aumento da letalidade no paciente internado por DCV atingiu a parcela mais economicamente ativa da população (20-59 anos), agregando mais uma preocupação à crise econômica vigente. Apesar de outros estudos observarem o impacto similar da pandemia no atendimento hospitalar, ^13 , 15 , 20^ nosso estudo talvez seja um dos primeiros a demonstrar um aumento da taxa de letalidade intra-hospitalar cardiovascular. ^12^

O estudo apresenta como limitação a avaliação apenas de uma parcela da população brasileira, a que tem acesso apenas ao SUS. Portanto, não se pode extrapolar tais dados para toda a população do país. É possível realçar que o impacto demonstrado na letalidade dos brasileiros esteja superestimado, uma vez que a população assistida pelo serviço público de saúde, sendo socialmente mais desprovida de recursos, apresenta pior controle dos fatores de risco cardiovasculares, além de acesso a medicação de qualidade inferior e de forma insatisfatória. ^24^ Tais fatores *per se* tornam-na mais vulnerável para descompensação clínica, com consequente maior letalidade intra-hospitalar. Ademais, a restrição de internações eletivas pode ter tido certa influência na letalidade intra-hospitalar, apesar desse tipo de atendimento representar apenas 7,80% do total de internações ( Tabela 1 ). É importante destacar o curto período analisado (3 meses). Novos estudos, portanto, devem validar esses achados ao compará-los com outros períodos.

## Conclusões

Nosso estudo é o primeiro a avaliar o impacto na saúde cardiovascular no SUS em todo o Brasil durante a pandemia da COVID-19. Esses dados fundamentam preocupações de que a assistência possa estar sendo adiada ou abreviada durante a pandemia da COVID-19.
