# Supplementary material for: Redução na Hospitalização e Aumento na Mortalidade por Doenças Cardiovasculares durante a Pandemia da COVID-19 no Brasil
Source: Arq Bras Cardiol. 2021 Feb 4;116(3):371–80. [Article in Portuguese] doi: 10.36660/abc.20200821 (PMC8159562; doi:10.36660/abc.20200821)
Supplement: Translation

# Translation: Reduction in Hospitalization and Increase in Mortality Due to Cardiovascular Diseases during the COVID-19 Pandemic in Brazil

**Authors:** Paulo Garcia Normando, José de Arimatéia Araujo-Filho, Gabriela de Alcântara Fonseca, Rodrigo Elton Ferreira Rodrigues, Victor Agripino Oliveira, Ludhmila Abrahão Hajjar, André Luiz Cerqueira Almeida, Edimar Alcides Bocchi, Vera Maria Cury Salemi, Marcelo Melo

**Affiliations:** 1 Brazil Universidade Federal da Paraíba, João Pessoa, PB - Brazil; 2 Brazil Universidade de São Paulo Instituto do Coração - Centro de Diagnóstico por Imagem, São Paulo, SP - Brazil; 3 Brazil Hospital Sírio Libanês, São Paulo, SP - Brazil; 4 Brazil Instituto de Coração – Cardiopneumologia, São Paulo, SP - Brazil; 5 Brazil Santa Casa de Misericórdia de Feira de Santana – Cardiologia, Feira de Santana, BA - Brazil

**Keywords:** COVID-19, Betacoronavirus, Pandemics, Cardiovascular Diseases/complications, Epidemiology, Hospitalization, Mortality, Comorbidities, Unified Health System

## Abstract

**Background:**

In the COVID-19 pandemic, the increase in the incidence of cardiovascular diseases (CVD) and mortality from them has been recognized worldwide. In Brazil, the impact of COVID-19 on CVD must be evaluated.

**Objectives:**

To assess the impact of the current pandemic on the numbers of hospital admissions (HA), in-hospital deaths (ID), and in-hospital fatality (IF) from CVD by use of national epidemiological data from the Brazilian Unified Public Health System.

**Methods:**

Time-series observational study using comparative analysis of the HA, ID, and IF due to CVD recorded from January to May 2020, having as reference the values registered in the same period from 2016 to 2019 and the values projected by linear regression methods for 2020. The statistical significance level applied was 0.05.

**Results:**

Compared to the same period in 2019, there was a 15% decrease in the HA rate and a 9% decrease in the total ID due to CVD between March and May 2020, followed by a 9% increase in the IF rate due to CVD, especially among patients aged 20-59 years. The HA and IF rates registered in 2020 differed significantly from the projected trend for 2020 (p = 0.0005 and 0.0318, respectively).

**Conclusions:**

During the first months of the pandemic, there were a decline in HA and an increase in IF due to CVD in Brazil. These data might have resulted from the inadequate planning of the CVD management during the pandemic. Thus, immediate actions are required to change this scenario. (Arq Bras Cardiol. 2021; [online].ahead print, PP.0-0)

## Introduction

The outbreak of the coronavirus disease 2019 (COVID-19) was declared a pandemic by the World Health Organization on March 11, 2020. In July 2020, Brazil ranked second in the number of cases and deaths from COVID-19. By July 24, 2020, Brazil had recorded 2 276 860 confirmed cases of COVID-19 and 84 551 deaths from the disease.^1^

Considering that COVID-19 is spread primarily via droplets expelled during talking, coughing, and sneezing, or via contaminated surfaces,^4^restrictions to people traffic and contact have been proposed by government and public health authorities in most western countries. In Brazil, the logistic challenges to meet the patients’ demands have included not only the increase in the number of beds in intensive care units and wards, but also the suspension of elective healthcare provision and of elective complementary tests and procedures, in addition to targeting public resources at the COVID-19 management.^5^

Population studies in other countries have reported a relative reduction in hospital admissions for cardiovascular diseases (CVD) during the COVID-19 pandemic,^8 , 9^in association with an increase in the fatality rates related to that group of diseases,^10 , 11^a reason for great concern in the international medical and scientific community.

Thus, we tested the hypothesis that, during the COVID-19 pandemic, there was a reduction in cardiovascular care provision and in the number of cardiovascular interventions performed, which might have led to higher in-hospital mortality from CVD in the general population. Using public databases from the Brazilian Unified Public Health System (SUS), this study aimed at assessing the impact of the pandemic on the number of hospital admissions and on in-hospital fatality due to CVD in Brazil from January to May 2020, as compared to those in the same months of the previous 4 years. In addition, pre-hospital clinical elements, such as elective procedures, were assessed.

## Methods

This is a time-series observational study to assess the hospital admissions, in-hospital deaths, and in-hospital fatality rates (percentage of deaths among the admissions) related to CVD at SUS own units or at healthcare units with which SUS maintains an agreement, from January to May of the years 2016 to 2020. In addition, CVD-related in-hospital and outpatient procedures performed at those units during the same periods were assessed. The first two variables were classified according to age group as follows: child/adolescent (0-19 years), adult (20-59 years), and elderly (60 years and older). Data were collected on July 9, 2020, from the Brazilian Hospital Information System and Brazilian Outpatient Information System of the SUS (SIH and SIA/SUS, respectively), available at the DATASUS platform. Those systems are public and anonymous, in accordance with the first article of the Resolution 510/2016 of the Brazilian Committee on Ethics and Research. It is worth noting that updates on past admissions can occur in the platform at any time, thus, it is not guaranteed that all data are consolidated, regardless of the year.

For the analysis of the procedures, we used the codes of the SUS System for the Management of the Table of Procedures, Medications, Orthoses, Prostheses and Materials (SIGTAP). For each procedure selected, the in-hospital and outpatient productions were added, considering all correlated procedure codes. The procedures were as follows:

Diagnostic procedures: cardiac catheterization, echocardiography (stress, transesophageal, and transthoracic), electrocardiography, cardiac pacemaker implantation, 24-hour Holter monitoring, ambulatory blood pressure monitoring (ABPM), exercise test.

Surgical procedures: cardiovascular, endovascular, and vascular surgeries.

To assess the number of admissions, in-hospital deaths, and in-hospital fatality, we selected, for each age group, the records of the secondary diagnoses related to the corresponding cardiovascular pathologies in the List of Morbidity of the 10th Revision of the International Statistical Classification of Diseases and Related Health Problems (CID-10). The following disease categories were considered: stroke, hypertensive diseases (essential arterial hypertension and other hypertensive diseases), rheumatic diseases (acute rheumatic fever and chronic rheumatic heart disease), acute myocardial infarction (AMI), heart failure, congenital malformations of the circulatory system, and conduction disorders and arrhythmias.

### Statistical Analysis

Considering that the procedures and surgeries are discriminated in the system regarding only their nature (outpatient or in-hospital), a descriptive analysis of how such procedures were distributed in those categories was performed. Regarding data from admission and in-hospital deaths, the variables sex, age group, skin color/race, and type of healthcare (urgency or emergency) were considered.

Aiming at understanding the possible impact of the pandemic on the dynamics of the procedures, admissions, and in-hospital deaths related to CVD, the numbers regarding the months of March, April, and May – the most affected months of 2020 by the pandemic in Brazil – were compared for the years 2016 to 2020. The values recorded from 2019 and 2020 and their corresponding percent variation were compared. It is worth noting that a variation in the number of admissions and in-hospital deaths from one year to the other or a change in the mean of the previous years as compared to 2020 is not necessarily caused by the pandemic. We considered this hypothetical variation a possible consequence of an already established trend in the previous years. Finally, the value expected for the year 2020 was estimated by use of linear regression. Such analysis allows assuming whether the number of procedures, surgeries, admissions, and in-hospital deaths or the in-hospital fatality rate observed in the previous years showed a trend towards increase or decrease. Thus, that analysis captured both the trend of the years and the statistical variations occurring in the previous years.

Although normality tests were not performed, the normal distribution of data over time was assumed, considering the central limit theorem, because the data of each year are a totalization of several random variables. Homocedasticity could not be completely verified, because DATASUS does not provide completely individualized data.

Statistical analyses were performed for both the number of surgeries and procedures, and for each individual procedure/surgery as well. Similarly, those analyses were performed for admissions, in-hospital deaths, and in-hospital fatality, considering each morbidity studied individually as well as the sum of all of them, which is an analysis of the cardiovascular causes in general.

Because linear regression has a gaussian error, Student *t* test was performed for the mean of a sample to compare the values projected with those recorded in 2020, and the null hypothesis was rejected with p<0.05 (95% confidence interval). The Microsoft® Excel® and Scilab® 6.1.0 software were used to perform the statistical analyses described and to build the tables and graphs.

## Results

### Descriptive Analysis

Based on the data collected regarding the months from January to May of the years 2016 to 2020, we identified 35 744 058 procedures, 1 336 472 hospital admissions, and 142 157 in-hospital deaths, and the last two were divided according to region, sex, age group, race/skin color, and healthcare type as shown in Table 1 . Complete data illustrating the variation in the numbers initiating in March 2020 are shown in the figures of the supplementary material.

**Table 1:** – Descriptive analysis of the number of procedures, surgeries, admissions, and in-hospital deaths from January to May of the years 2016 to 2020

	IN-HOSPITAL		OUTPATIENT	
	Quantity	Percentage	Quantity	Percentage
**Procedures**				
Adult cardiac catheterization	209 926	3.66%	252 162	0.87%
Echocardiography	989 776	17.24%	2 751 796	9.53%
Electrocardiography	4 461 799	77.71%	21 587 674	74.75%
Pacemaker implantation	48 666	0.85%	-	-
ABPM	-	-	2 763 748	9.57%
Holter monitoring	26 119	0.45%	513 471	1.78%
Exercise test	5 630	0.10%	1 010 144	3.50%
**Total**	**5 741 916**	**100.00%**	**28 878 995**	**100.00%**
**Surgeries**				
Cardiovascular	193 787	36.45%	-	-
Endovascular	48 024	9.03%	-	-
Vascular	289 815	54.51%	591 521	100.00%
**Total**	**531 626**	**100.00%**	**591 521**	**100.00%**
**Procedures**				
**Region**				
Northern	179 761	3.13%	1 442 382	4.99%
Northeastern	896 916	15.62%	5 551 764	19.22%
Southeastern	3 071 586	53.49%	15 271 343	52.88%
Southern	1 236 067	21.53%	4 285 803	14.84%
West-central	357 586	6.23%	2 327 703	8.06%
**Brazil**	**5 741 916**	**100.00%**	**28 878 995**	**100.00%**
**Surgeries**				
**Region**				
Northern	13 676	2.57%	13 774	2.33%
Northeastern	98 475	18.52%	442 540	74.81%
Southeastern	239 747	45.10%	81 777	13.82%
Southern	140 536	26.44%	43 469	7.35%
West-central	39 192	7.37%	9 961	1.68%
**Brazil**	**531 626**	**100.00%**	**591 521**	**100.00%**
	**ADMISSIONS**		**DEATHS**	
	**Quantity**	**Percentage**	**Quantity**	**Percentage**
**Morbidity**				
Stroke	357 040	26.72%	52 239	36.75%
Hypertensive diseases	153 048	11.45%	2 920	2.05%
Rheumatic diseases	19 125	1.43%	1 329	0.93%
Acute myocardial infarction	242 143	18.12%	24 753	17.41%
Heart failure	399 416	29.89%	43 906	30.89%
Congenital malformations	33 939	2.54%	2 370	1.67%
Conduction disorders and arrhythmias	131 761	9.86%	14 640	10.30%
**Total**	**1 336 472**	**100.00%**	**142 157**	**100.00%**
**Region**				
Northern	77 577	5.80%	8 578	6.03%
Northeastern	327 515	24.51%	35 749	25.15%
Southeastern	563 293	42.15%	63 609	44.75%
Southern	270 995	20.28%	23 624	16.62%
West-central	97 092	7.26%	10 597	7.45%
**Total**	**1 336 472**	**100.00%**	**142 157**	**100.00%**
**Sex**				
Male	704 163	52.69%	73 227	51.51%
Female	632 309	47.31%	68 930	48.49%
**Total**	**1 336 472**	**100.00%**	**142 157**	**100.00%**
**Race/Skin color**				
White	491 422	36.77%	48 962	34.44%
Black	58 968	4.41%	6 301	4.43%
Mixed	435 848	32.61%	46 276	32.55%
Yellow	28 768	2.15%	2 764	1.94%
Native	1 025	0.08%	107	0.08%
No information	320 441	23.98%	37 747	26.55%
**Total**	**1 336 472**	**100.00%**	**142 157**	**100.00%**
**Age group (years)**				
0-19	44 658	3.34%	3 241	2.28%
20-59	415 122	31.06%	29 245	20.57%
60+	876 692	65.60%	109 671	77.15%
**Total**	**1 336 472**	**100.00%**	**142 157**	**100.00%**
**Type of care**				
Elective	104 229	7.80%	5 454	3.84%
Urgent	1 232 243	92.20%	136 703	96.16%
**Total**	**1 336 472**	**100.00%**	**142 157**	**100.00%**

*ABPM: ambulatory blood pressure monitoring.*

### Graph Analysis

1A shows the data regarding the diagnostic and surgical procedures performed from March to May of 2016 to 2020. In addition, the estimates for 2020 (dotted lines), calculated by using data from 2016 to 2019, are also shown. A trend towards an increase in the number of diagnostic and surgical procedures (dotted lines) for the year 2020 is observed. However, the real data show a significant decrease when compared to data from the previous year.

Figure 1B depicts the numbers of admissions and in-hospital deaths recorded, considering all morbidities studied. In addition, the estimates for 2020 (dotted lines), calculated by using data from 2016 to 2019, are also shown. The graph shows the same trend towards an increase observed in the previous graph but not confirmed in the year 2020, when a steep decline is observed. Regarding the number of deaths, the projected trend would be that of maintenance, contrasting with the intense reduction in the number of in-hospital deaths recorded in the months of March to May 2020.

Figure 1C illustrates the in-hospital fatality rate due to CVD in general, showing a drastic increase in the in-hospital fatality rate in 2020 as compared to those recorded in previous years. In this case, the variation is in the opposite direction to that observed for the admissions and in-hospital deaths, which decreased in 2020.

**Figure 1:** – Analysis of the trend in: (a) the number of procedures and surgeries; (b) the number of admissions and deaths; and (c) in-hospital fatality rate in March to May of 2016 to 2020. p-value calculated from the difference between the value projected and the value recorded in 2020 using Student t distribution.

That analysis was replicated for each type of procedure and surgery, as well as for the number of admissions, in-hospital deaths and in-hospital fatality rate for each pathology studied. Those results are summarized in Tables 2 and 3 , which show data from 2019 and 2020, the percent difference between these years, the value projected for 2020 (which indicates the trend from 2016 to 2019), the confidence interval, and the p-value of that projection. The results according to age group are shown in the supplementary material.

**Table 2:** – Statistical analysis of the reduction in the number of procedures and surgeries in March to May of 2019 and 2020 and their comparison

	Number of procedures		Percent difference 2020 - 2019	Number of procedures estimated for 2020 (March-May)	Confidence interval (95% CI)	p-value
	2019 March-May	2020 March-May				
**Procedures**						
Adult cardiac catheterization	61 502	44 652	-27%	63 823	68 921 – 58 724	0.0041
Echocardiography	530 448	307 221	-42%	568 014	599 663 – 536 364	0.0009
Electrocardiography	3 624 680	2 153 969	-41%	3 795 712	3 952 567 – 3 638 856	0.0005
Pacemaker implantation	5 993	5 315	-11%	6 129	6 711 – 5 546	0.0276
ABPM	498 923	127 730	-74%	609 445	748 875 – 470 014	0.0048
Holter monitoring	79 792	38 815	-51%	84 592	93 971 – 75 212	0.0024
Exercise test	137 678	56 501	-59%	134 673	149 970 – 119 375	0.0022
** All**	**4 939 016**	**2 734 203**	**-45%**	**5 262 388**	**5 516 598 – 5 008 177**	**0.0006**
**Surgeries**						
Cardiovascular	23 907	20 744	-13%	24 045	26 897 – 21 191	0.0388
Vascular	101 694	83 786	-18%	104 654	159 586 – 49 720	0.1914
Endovascular	37 547	25 461	-32%	38 208	45 145 – 31 269	0.0165
** All**	**163 148**	**129 991**	**-20%**	**166 906**	**218 270 – 115 540**	**0.0854**
**TOTAL**	**5 102 164**	**2 864 194**	**-44%**	**5 429 294**	**5 670 710 – 5 187 876**	**0.0005**

*ABPM: ambulatory blood pressure monitoring. Confidence intervals and p-values calculated by using Student t distribution, considering the differences between the value projected and the value recorded in 2020.*

**Table 3:** – Statistical analysis of the number of admissions, in-hospital deaths, and in-hospital fatality rate in March to May of 2019 and 2020 and their comparison

	Data recorded in		Percent difference 2020 - 2019	Estimates for 2020 March-May	Confidence interval (95% CI)	p-value
	2019 March - May	2020 March - May				
**Admissions**						
Stroke	45 214	39 900	-12%	46 199	48 586 – 43 811	0.0082
Hypertensive diseases	18 278	12 229	-33%	18 053	20 331 – 15 773	0.0088
Rheumatic diseases	2403	1701	-29%	2 266	2683 – 1847	0.0294
Acute myocardial infarction	31 566	30 298	-4%	33 084	35 550 – 30 616	0.0405
Heart failure	47 250	39 667	-16%	46 077	49 847 – 42 305	0.0191
Cardiovascular malformations	4489	3692	-18%	4602	4883 – 4320	0.0055
Conduction disorders and cardiac arrhythmias	16 875	13 977	-17%	17 212	18 316 – 16 107	0.0067
**Cardiovascular comorbidities**	**166 075**	**141 464**	**-15%**	**167 491**	**169 772 – 165 209**	**0.0005**
**In-hospital deaths**						
Stroke	6411	5871	-8%	6363	7364 - 5361	0.1439
Hypertensive diseases	336	289	-14%	309	361 - 256	0.1926
Rheumatic diseases	180	144	-20%	161	262 - 58	0.3414
Acute myocardial infarction	3081	2805	-9%	3094	3500 - 2686	0.0872
Heart failure	5356	4845	-10%	5212	5612 - 4811	0.0579
Cardiovascular malformations	307	288	-6%	297	382 - 210	0.3995
Conduction disorders and cardiac arrhythmias	1952	2053	5%	1986	2582 - 1388	0.3863
** Cardiovascular comorbidities**	**17 623**	**16 295**	**-8%**	**17 420**	**19 062 – 15 777**	**0.0918**
**In-hospital fatality rate**						
Stroke	14.18	14.71	4%	13.72%	15.33 - 12.10	0.1067
Hypertensive diseases	1.84	2.36	29%	1.72%	2.11 - 1.34	0.0198
Rheumatic diseases	7.49	8.47	13%	7.08%	10.19 - 3.97	0.1616
Acute myocardial infarction	9.76	9.26	-5%	9.20%	10.21 - 8.18	0.4363
Heart failure	11.34	12.21	8%	11.31%	12.01 - 10.60	0.0322
Cardiovascular malformations	6.84	7.80	14%	6.40%	8.26 - 4.54	0.0798
Conduction disorders and cardiac arrhythmias	11.57	14.69	27%	11.57%	14.90 - 8.24	0.0559
** Cardiovascular comorbidities**	**10.61**	**11.52**	**9%**	**10.39%**	**11.26 - 9.52**	**0.0318**

*Confidence intervals and p-values calculated by using Student t distribution, considering the differences between the value projected and the value recorded in 2020.*

#### Diagnostic and Surgical Procedures

Table 2 shows the comparison of the number of diagnostic and surgical procedures performed in March, April, and May of 2019 and 2020, with a total drop of 45% in all procedures studied in 2020. The procedures with the most significant reductions were as follows: ABPM (74% reduction), exercise test (59%), and 24-hour Holter (51%). Electrocardiography and echocardiography had a decrease of 41% and 42%, respectively. Cardiac catheterization and pacemaker implantation had the smallest decline, 27% and 11%, respectively.

The total number of surgeries performed in March, April, and May 2020 decreased by 20% as compared to the previous year, which was not statistically significant (p=0.0854). However, when considering only cardiovascular and endovascular surgeries, declines of 13% and 32%, respectively, were observed, both with statistical significance (p<0.05).

## Admissions

Regarding the admissions due to cardiovascular diseases in March, April, and May 2020, a 15% reduction was observed as compared to the same period of the previous year ( Table 3 ). Data from all diseases analyzed individually also showed a statistically significant reduction. The greatest differences were in admissions due to hypertensive diseases, followed by those due to rheumatic diseases, with 33% and 29% reductions, respectively. It is worth noting that admissions due to AMI had the smallest reduction (4%).

In general, all diseases showed a decrease in admissions from 2019 to 2020 for all age groups. The following are worthy of note: AMI, whose difference in admissions had statistical significance only for the elderly group; heart failure, in which the extreme age groups (child/adolescent and elderly) had the greatest impacts; and conduction disorders and other arrhythmias, which showed statistically significant reduction only for the elderly. When considering admissions from stroke, the adults and the elderly had significant reductions (11% and 12%, respectively). Regarding admissions from hypertensive diseases, all age groups had statistically significant reductions.

## In-hospital Deaths and In-hospital Fatality Rate

The absolute number of deaths due to CVD decreased by 8% from March to May 2020 as compared to the same period in 2019 ( Table 3 ). The deaths related to hypertensive diseases in the age group 20-59 years had a 21% increase in 2020 (p<0.05; supplementary material).

Regarding the general fatality rates, there was an overall 9% increase when comparing the same months of 2019 and 2020. Except for AMI, whose fatality rate decreased by 5%, all other pathologies had an increase in fatality rates. Regarding the pathologies individually, hypertensive diseases and heart failure stood out, with 29% and 8% increases in their in-hospital fatality rates, respectively, from 2019 to 2020 (p<0.05).

When considering the age groups, the in-hospital fatality increase in the admissions due to CVD was statistically significant only among adults. Considering the diseases individually, it is worth noting that the in-hospital fatality increase due to hypertensive diseases was statistically significant only for adults. Regarding heart failure, adults and elderly showed statistical difference in that rate (p<0.05; Tables 2 and 3 of the supplementary material).

## Discussion

This study shows a reduction in the cardiovascular care provided to the Brazilian population by the SUS during the COVID-19 pandemic, which resulted in both a reduction in the number of hospital admissions for CVD and an increase in the in-hospital fatality rate from those diseases.

Our results are similar to those of a study carried out in Italy during 7 days in March 2020, which showed a 13.3% reduction in the proportion of patients with AMI as compared to that same week of 2019. In addition, that study reported a 39.2% increase in acute coronary syndromes and a 31.5% increase in the time elapsed from medical contact to coronary revascularization.^12^

In our study, regarding the number of admissions, there was a reduction for all morbidities and age groups analyzed, mainly in April and May, possibly because of the COVID-19 pandemic. Other studies carried out in different countries have reported similar findings.^13^The population’s fear of contracting the virus and the systematization of the healthcare, prioritizing the pandemic, justify that initial impact.^16 , 17^That reduction has also been reported for other diseases, as shown by the studies on stroke in Italy^18^and China.^19^

The reassignment of human resources in the fight against COVID-19 was similar in several countries, despite the heterogeneity of their health systems.^12^Some countries, such as Australia and New Zealand, to prepare hospitals to provide healthcare to a large number of patients with COVID-19, kept the delivery of surgical care limited to emergency or high-priority elective cases.^20^In a hospital in Northern Italy, one of the epicenters of the pandemic in that country, the planned surgical activities were interrupted to increase the number of intensive care physicians available to patients with COVID-19, and the outpatient activities were cut to half.^21^This rearrangement in the healthcare model was expected to have an impact on the mortality from other diseases, whose usual healthcare delivery flow was reduced, favoring clinical decompensations, diagnostic delay, and disease progression.

Despite the reduction in absolute numbers of in-hospital deaths, there was an increase in the in-hospital fatality rate of admissions due to CVD. That reduction in the number of deaths might have resulted from the lack of proper reporting and the deficient structure of the health system to properly designate the COVID-19-related cause of death as cardiovascular. The interaction between COVID-19 and the cardiovascular system is currently well known, after 7 months of disease. COVID-19 is related to a high prevalence of cardiac injury, arrhythmias, myocarditis, acute coronary syndrome, heart failure, cardiogenic shock, and thromboembolic events.^22^The increased fatality in admissions due to CVD reflects the potential severity of COVID-19 in CVD and the possible patient’s delay in searching medical care, being then hospitalized in more severe conditions. The increased fatality of patients admitted due to CVD has reached the most economically-active part of the population (20-59 years), adding more concern to the ongoing economic crisis. Although some studies have observed a similar impact of the pandemic on hospital care,^13 , 15 , 20^ours might be one of the first to demonstrate an increase in the in-hospital fatality rate due to CVD.^12^

This study has the limitation of assessing only one part of the Brazilian population, the one with access to only the SUS-provided healthcare. Thus, our data cannot be extrapolated to the whole population of Brazil. It is worth noting that the impact demonstrated on the fatality of Brazilians might be overestimated, because the population cared for at the SUS, being socioeconomically disadvantaged, has a poorer control of cardiovascular risk factors, in addition to having access to medication of poorer quality and in an unsatisfactory manner.^24^Such factors on their own make that population more vulnerable to clinical decompensation, with consequent higher in-hospital fatality. Moreover, the restriction of elective admissions might have influenced in-hospital fatality, although that type of healthcare represents only 7.80% of the total of admissions ( Table 1 ). It is worth noting the shortness of the period analyzed (3 months). Therefore, further studies should validate these findings and compare them to those of other periods.

## Conclusions

Ours is the first study to assess the impact on cardiovascular health at the SUS across Brazil during the COVID-19 pandemic. These data support the concern that healthcare delivery might have been postponed or reduced during the COVID-19 pandemic.
